# Long-read sequencing settings for efficient structural variation detection based on comprehensive evaluation

**DOI:** 10.1186/s12859-021-04422-y

**Published:** 2021-11-12

**Authors:** Tao Jiang, Shiqi Liu, Shuqi Cao, Yadong Liu, Zhe Cui, Yadong Wang, Hongzhe Guo

**Affiliations:** Faculty of Computing, Harbin Institute of Technology, Harbin, 150001 China

**Keywords:** Long-read sequencing, SV calling, Coverage, Read length, Sequencing error, Comprehensive evaluation

## Abstract

**Background:**

With the rapid development of long-read sequencing technologies, it is possible to reveal the full spectrum of genetic structural variation (SV). However, the expensive cost, finite read length and high sequencing error for long-read data greatly limit the widespread adoption of SV calling. Therefore, it is urgent to establish guidance concerning sequencing coverage, read length, and error rate to maintain high SV yields and to achieve the lowest cost simultaneously.

**Results:**

In this study, we generated a full range of simulated error-prone long-read datasets containing various sequencing settings and comprehensively evaluated the performance of SV calling with state-of-the-art long-read SV detection methods. The benchmark results demonstrate that almost all SV callers perform better when the long-read data reach 20× coverage, 20 kbp average read length, and approximately 10–7.5% or below 1% error rates. Furthermore, high sequencing coverage is the most influential factor in promoting SV calling, while it also directly determines the expensive costs.

**Conclusions:**

Based on the comprehensive evaluation results, we provide important guidelines for selecting long-read sequencing settings for efficient SV calling. We believe these recommended settings of long-read sequencing will have extraordinary guiding significance in cutting-edge genomic studies and clinical practices.

**Supplementary Information:**

The online version contains supplementary material available at 10.1186/s12859-021-04422-y.

## Background

Structural variation (SV) is a fundamental genomic alteration that usually refers to a change of over 50 bp nucleotide fragments, including insertion, deletion, inversion, translocation and duplication [1]. A considerable number of studies have confirmed that there are approximately 20,000 SVs in each person [2–5], which contribute to the diversity and evolution of human genomes at both the individual and population levels [6]. These SVs significantly affect molecular and cellular processes and regulatory functions, are associated with complex phenotypes [7, 8], such as autism [9] and schizophrenia [10], and they have been implicated in genomic diseases such as Mendelian disorders and cancers.

Recently, a large number of SVs have been detected through long-read sequencing technologies, such as Pacific Biosciences (PacBio) [11] and Oxford Nanopore Technologies (ONT) [12], which can produce reads of approximately 10 kbp or even up to 2 Mbp. With the ever-rising read length, long-read sequencing enables the unraveling of twofold more SVs than short-read sequencing efforts [8, 13]. In particular, due to the increase in mappability based on excellent long-range spanning information, it is possible to collect variant evidence across tens to thousands of kilobases [14] and discover large and complex SVs, particularly in repetitive genomic regions [15]. With these advancements, long-read sequencing technologies have become the most effective tool for revealing the full spectrum of genetic variation, improving the understanding of mutation and evolutionary processes, resolving some of the missing heritability, and helping to discover more novel biological insights [16].

Although new methods have evolved to apply long reads for the accurate and sensitive discovery of SVs, fundamental differences among attributes across various long-read sequencing platforms impact the effect, cost, and time for the identification of SVs. Because of the distinction in preparing DNA fragments and applying chemical techniques, different platforms usually generate diverse types of data, such as PacBio continuous long reads (CLR), PacBio circular consensus sequencing [17], ONT long reads and ultra-long reads [12]. The major differences between these types of long reads are their read length and error rate, e.g., the typical length range and accuracy for CLR are 5–60 kbp and 85–92% [18–21], for CCS they are 10–30 kbp and over 99% [17], for ONT long reads they are 10–100 kbp and 87–98% [16], and for ONT ultra-long reads they are greater than 100 kbp and 87–98%[22, 23]. To a certain extent, the longer read length can contribute to the detection of larger SVs, and the lower error rate improves accuracy in the prediction of SV breakpoints and size, especially for intermediate-size SVs less than 2 kbp. As another key point across platforms, sequencing coverage is an essential attribute for the discovery of heterozygous SVs, and higher coverage will make the process of detection more accurate and sensitive. However, compared with short reads, long reads are still relatively low throughput and more expensive. To be more specific, the estimated cost per Gb for CLR and CCS generated from the latest platform of Sequel II are $13–$26 and $43–$86, respectively; for ONT PromethION, it is $21–$42, so even for ONT, ultra-long read have an estimated total cost of $500–$2000 [16]. In contrast, the cost per Gb for short reads has decreased to less than $10 [24]. The expensive cost for long-read data with higher coverage will greatly limit widespread adoption. Therefore, there is an urgent need to establish guidelines for sequencing coverage, read length, and error rate that ensure acceptable high SV yields and that achieve the lowest cost at the same time.

To address this issue, we generated a full range of simulated error-prone long-read datasets containing various sequencing settings as baselines and comprehensively evaluated the performance of SV calling with state-of-the-art long-read alignment-based methods as feedback for the selection of the best sequencing settings. The benchmark results show that the overall F1 score and Matthews correlation coefficient (MCC) [25] rate increase along with the coverage, read length, and accuracy rate. Notably, it is sufficient for sensitive and accurate SV calling in practice when the long-read data comes to 20× coverage, 20 kbp average read length, and approximately 10–7.5% or below 1% error rates (or approximately 90–92.5% or over 99% accuracy rate). There is no significant optimization of SV calling even if the attributes are further improved, even though a greater cost of sequencing is required. With adequate assessment, we provide recommendations regarding the long-read sequencing settings on the coverage, mean read length, and error rate that achieve better sequencing economy and effectiveness of SV detection, and this will play an important role in future research work for SV detection based on long-read sequencing and will have extraordinary guiding significance.

## Results

### The overview of this study

We produced abundant simulated error-prone long-read datasets with diverse sequencing settings and comprehensively evaluated the performance on SV calling with a series of state-of-the-art long-read-based SV callers. Three major steps of this approach are as follows.

- Step 1: Generate simulated synthetic diplontic long-read datasets based on various sequencing attributes (i.e., sequencing coverage, read length, and error rate).
- Step 2: Discover structural variants using the state-of-the-art SV methods and an ensemble method.
- Step 3: Comprehensively evaluate SV calling performance on various sequencing datasets to compute the better sequencing settings for achieving satisfactory performance.

**Fig. 1:**
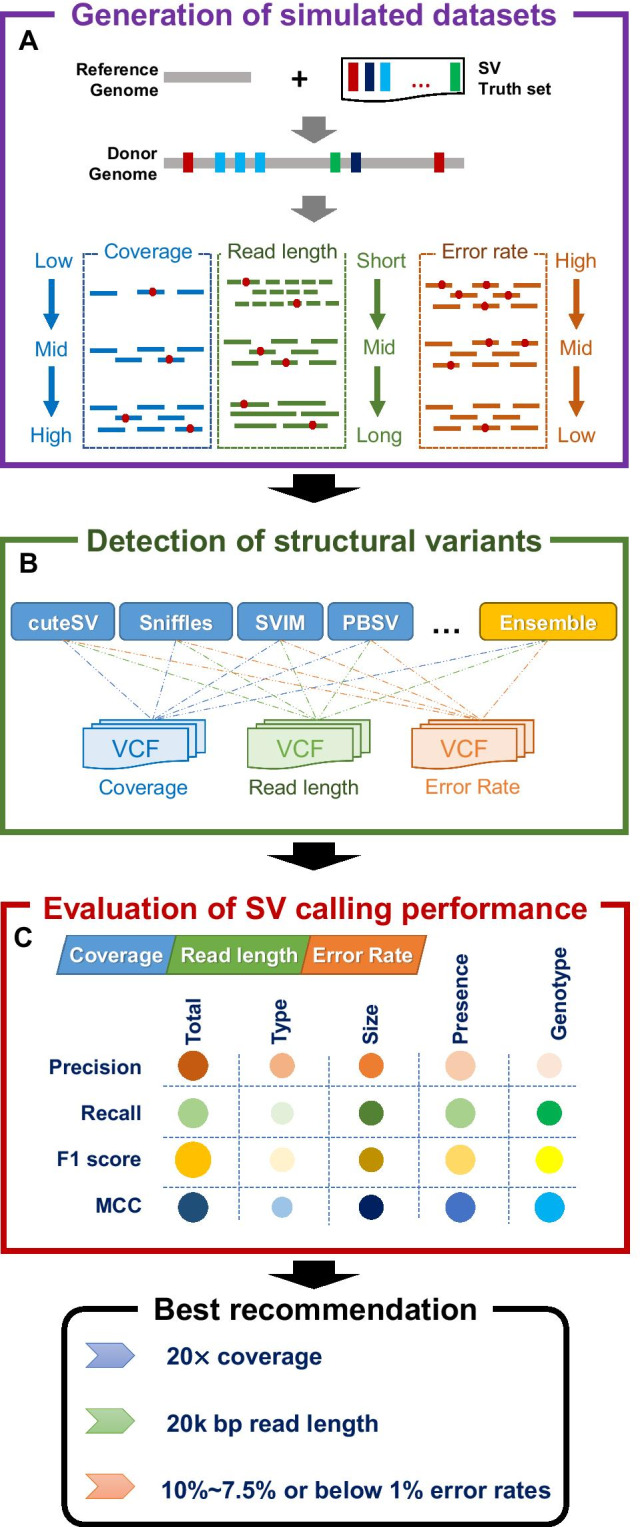
The overall workflow of the study. **a** Generation of simulated synthetic diplontic long-read datasets. **b** Detection of structural variants using various SV callers and an ensemble method. **c** Comprehensive evaluation of SV calling performance for the given sequencing setting guideline

The complete workflow of the study is shown in Fig. 1, and more details are provided in the Methods section.

### Evaluation of the sequencing coverage impact on SV calling

**Fig. 2:**
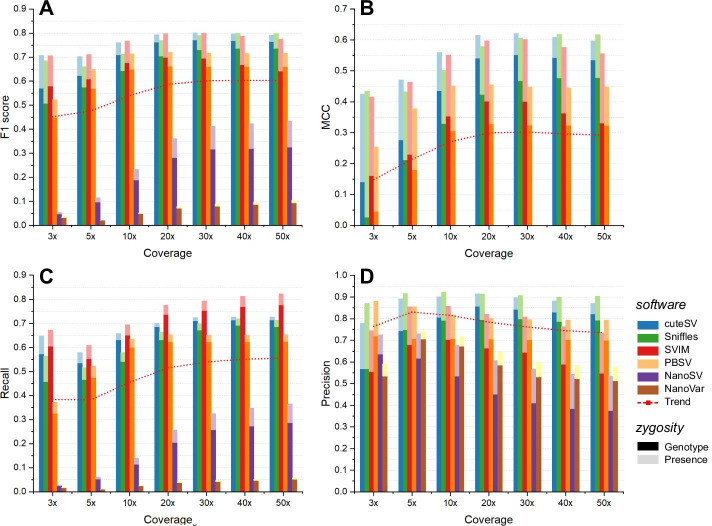
Comparison of the SV calling performance under diverse sequencing coverages. **a** The F1 score, **b** MCC, **c** recall rate and **d** accuracy of each tool. Dark and light colors indicate SV calling considering genotype and only presence. The red dotted line in each diagram indicates the overall trend with the sequencing depths

We first benchmarked the SV calling performance on 3×, 5×, 10 ×, 20×, 30×, 40×, and 50× sequencing depth datasets (20 kbp average read length and 10% error rate) to establish a baseline assessment. We selected six state-of-the-art SV callers, i.e., cuteSV [26], NanoSV [27], NanoVar [28], Sniffles [4], SVIM [29], and PBSV (https://github.com/PacificBiosciences/pbsv), to represent the ability of SV detection at different sequencing depths. The results in Fig. 2 and Additional file 1: Table S1 show that the average F1 score increased along with sequencing coverage, and approximately 0.152 in total grew from 3× to 50×. MCC kept pace with the F1 score and increased by 0.143 at the same coverage range. Especially in regard to 20×, the best three SV callers achieved brilliant resolution, i.e., cuteSV (F1: 0.800, MCC: 0.620), SVIM (F1:0.798, MCC: 0.597), and Sniffles (F1:0.769, MCC: 0.578), which indicates that a relatively low depth of 20× enables SV calling approaches to obtain high performance (Additional file 1: Table S2). Similarly, the recall rate also increased along with sequencing coverage, and improved by 0.173 overall from 3× to 50× . The best three tools mentioned above surpassed 0.65 recall rates on 20× sequencing data as well, i.e., cuteSV 0.719, SVIM 0.776, and Sniffles 0.664 (Additional file 1: Table S1-2). However, the precision decreased as the sequencing depth rose and declined by 0.095 overall from 5× to 50× . On the whole, the higher coverage data could greatly promote SV callers to uncover more SVs, although it would sacrifice a little precision.

To obtain a reliable minimum estimation of sequencing coverage, we computed the trend curve between F1 scores and sequencing depths for each method. All predicted trend curves were in the form of quadratic polynomials and are shown in Additional file 1: Table S3. Generally, the simulated long-read data in 30× to 40× could be the best coverage setting for SV calling methods that enabled them to achieve their best F1 scores; e.g., cuteSV, Sniffles and SVIM obtained 0.80 F1 scores under coverage of 34×, 41×, and 32×, respectively. In addition, a correlation coefficient over 0.92 indicates the high reliability of the predictions. More importantly, it is evident that 20× is sufficient for those tools with a brilliant resolution to complete SV calling; e.g., cuteSV, Sniffles, and SVIM each achieved an F1 score over 0.75.

Then, we assessed the impact of sequencing coverage on various SV types of calls, and the results are shown in Additional file 1: Figure S1A to S1D. There were more deletions and insertions accurately detected on deep coverage datasets, and the F1 score increased by 0.170 and 0.148 totally from 3× to 50×, respectively. The F1 scores of duplication and inversion also grew (i.e., 0.041 and 0.016, respectively), whereas the increments were not significant compared with deletion and insertion (Additional file 1: Table S1). This was mainly due to the greater complexity and larger size of duplicated and inverted calls in our ground truth sets. Discovering these SVs was more dependent on the improvement of read length and sequencing accuracy. Furthermore, almost all types of SVs achieved satisfactory performance on the 20× dataset, especially in detecting duplications after the apparent existence of a peak at approximately 20× (Additional file 1: Figure S1C).

We also evaluated the impact of sequencing coverage on six size scales of calls: 50–99 bp, 100–499 bp, 500–1 kbp, 1 k–5 kbp, 5 k–10 kbp, and > 10 kp. From the results shown in Additional file 1: Figures S1E to S1J, it is evident that the higher the sequencing depth was, the greater the F1 score was obtained, and the growth rate of F1 slowed down significantly on each dataset over 20× (Additional file 1: Table S2). On the other hand, SVs with large size were more dependent on higher coverage datasets compared with small and medium size SVs. It also makes sense that higher coverage data could contain more reads with a longer length and provide more significant signals for larger SVs.

In addition, we further benchmarked the performance of SV calling involving genotypes to determine the sequencing coverage impact in more detail (Fig. 2). Performance remained the same for each approach between identifying genotyped SV (SV with genotype) calls and ungenotyped SV (SV without genotype) calls; that is, higher coverage simulated data could promote the growth of F1 score in calling genotyped SVs. In addition, 20× is still a recommendable sequencing depth setting for the detection of genotyped SVs since SV callers such as cuteSV achieved scores of over 0.761 F1 at this coverage (Additional file 1: Table S2). Moreover, we calculated the difference in F1 score for each SV caller between their genotyped and ungenotyped SV calls. The F1 scores for SV calls that involve genotypes were approximately 0.04 ~ 0.12 lower than those with presence-only SVs (Additional file 1: Table S2). Hence, genotyping remains a large challenge for SV calling even at high sequencing coverage.

### Evaluation of the read length impact on SV calling

**Fig. 3:**
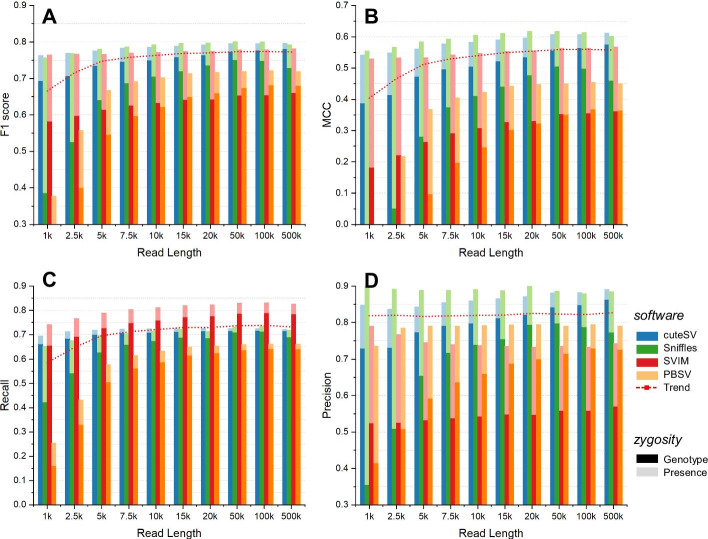
Comparison of the SV calling performance under diverse read lengths. **a** The F1 score, **b** MCC, **c** recall rate and **d** accuracy of each tool. Dark and light colors indicate SV calling considering genotype and only presence. The red dotted line in each diagram indicates the overall trend with the read lengths

We then applied 10 simulated long reads, including 1 kbp, 2.5 kbp, 5 kbp, 7.5 kbp, 10 kbp, 15 kbp, 20 kbp, 50 kbp, 100 kbp, and 500 kbp in read length (50× coverage and 10% sequencing error rate), for SV callers to evaluate the ability of SV detection. In this benchmark, we excluded NanoSV and NanoVar due to their poor performance. The results are shown in Fig. 3 and Additional file 1: Table S4-5. It is evident that the F1 score increased along with the read length, and approximately 0.107 increased from 1 to 500 kbp. Evidently, almost all tools performed well using datasets with read lengths from 20 to 50 kbp. Considering the higher cost for the longer reads, 20 kbp is a promising choice and is sufficiently capable to detect the SVs accurately and sensitively; e.g., cuteSV and Sniffles had an F1 score surpassing 0.79, SVIM followed closely with approximately 0.78, and the average F1 score of four SV callers was approximately 0.770. The MCC rate was consistent with F1 and increased with increasing read length. On 20 kbp, the average MCC was 0.554, which indicated a moderately positive linear relationship. With increasing read length, the recall rate improved by approximately 0.146 from 1 to 500 kbp, and on 20 kbp, cuteSV and Sniffles surpassed 0.71 SVIM performed better by approximately 0.82. However, the precision was almost stable with respect to read length and achieved an average of 0.821 in total. To a certain extent, a higher read length evidently increased the F1 score and recall and even slightly improved the precision. However, a horizontal comparison with coverage shows that the improvement in the ability of SV detection under different lengths was not as much as that for coverages, which indicates that coverage would have a greater impact than read length. When we fitted the trend curve of each tool under different read lengths, the correlation coefficient was not sufficiently qualified to predict the best recommendation in our specific simulated datasets. It was necessary to conduct more in-depth analysis for the best prediction of read length using each tool under more introduced SVs (Additional file 1: Table S3).

We then assessed the impact of read length on various SV types of SV calling. From Additional file 1: Figures S2A to S2C, the F1 scores in detecting deletions, insertions and duplications had almost the same increase of approximately 0.1; when data with a 500 kbp mean read length were applied, the F1 scores reached 0.839, 0.741, and 0.616, respectively (Additional file 1: Table S4). When using 20 kbp read length data, the performance of the detection on the three types mentioned above was also satisfactory; i.e., F1 scores were approximately 0.844, 0.738, and 0.530, respectively. However, the increase in F1 scores on inversions was not evident (Additional file 1: Figure S2D). In other words, read length greatly influenced deletion, insertion, and duplication calling but not inversion calling.

For different sizes of SVs, the results were similar to sequencing coverage in that the longer the read length was, the higher the F1 score was. Specifically, from Additional file 1: Figures S2E to S2J, the F1 scores of the six scales could increase by 0.103, 0.103, 0.161, 0.256, 0.350, and 0.055 (Additional file 1: Table S5). It is evident that the larger the SVs were, the greater the need for read length. These improvements were mainly because reads with longer lengths had greater potential to completely cover the region of SV and provide clearer SV signatures for discovering larger SVs. However, this rate of increase declined, as longer read length also decreases the yield of data, reducing the absolute number of reads containing SV signatures. Although the improvement of SV over 10 kbp was not as apparent as SV with other scales, the average F1 score surpassed 0.773, which indicated that the detection of longer SV was more advanced. Moreover, with the 20 kbp read length, the detection of each SV length scale had good performance, and the F1 scores of the six scales were 0.675, 0,770, 0.742, 0.789, 0.746, and 0.790 (Additional file 1: Table S5). Overall, the 20 kbp dataset could lead to satisfactory performance under different length scales.

When considering genotypes, simulated data with a longer read length could increase the F1 score significantly (Fig. 3). Similarly, a 20 kbp read length was still a satisfactory setting for detection; e.g., cuteSV reached a 0.765 F1 score by using this read length (Additional file 1: Table S5). After calculating the difference of F1 scores between considering genotypes and ignoring genotypes, we found that the F1 scores for SV calls that considered genotypes were approximately 0.03–0.14 smaller than those of SV calls that were only considering presence (Additional file 1: Table S4), and with the increase of read length, the differences between them were becoming smaller. All the above results show that longer reads could help genotyping consistency.

### Evaluation of the error rate impact on SV calling

**Fig. 4:**
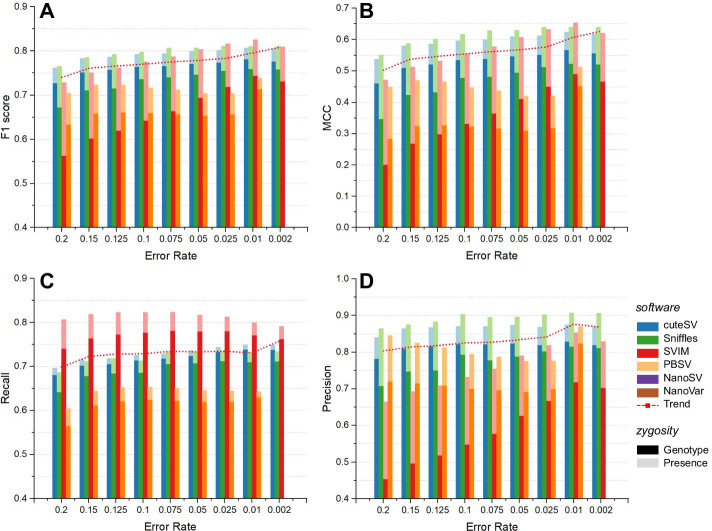
Comparison of the SV calling performance under diverse error rates. **a** The F1 score, **b** MCC, **c** recall rate and **d** accuracy of each tool. Dark and light colors indicate SV calling considering genotype and only presence. The red dotted line in each diagram indicates the overall trend with the sequencing error rates. It is worth noting that the average performance in 0.2% error data only consists of cuteSV, Sniffles and SVIM, and PBSV was excluded due to its relatively poor results

In this section, we applied nine simulated datasets, including 20%, 15%, 12.5%, 10%, 7.5%, 5%, 2.5%, 1%, and 0.2% error rates (50× sequencing coverage and 20 kbp read length) to evaluate the ability of the SV callers to detect various kinds of SVs with high performance. It should be noted that the data with 1% and 0.2% sequencing errors were produced by the consensus circular sequencing (CCS, also known as ‘HiFi’) model to make the datasets more consistent with the real situation of highly accurate long high-fidelity reads, and the 0.2% error rate dataset represented the latest HiFi reads [17]. From Fig. 4 and Additional file 1: Table S6, we found that the F1 score increased by approximately 0.073 from a 20 to 0.2% error rate. Notably, there was a sharp improvement around the 1% error rate (i.e., average 0.796 F1 score), which demonstrated that the datasets using the CCS/HiFi model showed more potential in making the SV callers perform much better than the others. Apart from CCS/HiFi reads, the F1 score and MCC increased along with a decrease in sequencing errors from 20 to 2.5%. In regard to 10–7.5% error rates, all four SV callers could obtain a brilliant performance; e.g., Sniffles could reach a 0.8 F1 score and cuteSV could surpass a 0.79 F1 score (Additional file 1: Table S7). The recall rate had no significant increase and maintained an average of 0.730. However, we found that the precision increased relatively noticeably as the error rate decreased; to be more specific, at a 20% error rate, the precision was only 0.804, but it surpassed 0.87 at 1% and 0.2% error rates, which indicates that SV callers had a competent ability to detect SV more precisely on more accurate long reads. Reducing the error rate could significantly improve the precision on the premise of ensuring the recall, and then the F1 score could also be improved. Although the overall performance was improved, the improvement was not nearly as much as that from changing the sequencing coverage and read length.

The trend curves in Additional file 1: Table S3 showed that using long reads with sequencing accuracy rates of approximately 1.04 and 1.00 could make cuteSV and Sniffles achieve their best F1 scores approximately 0.81. Although with the highly effective correlation coefficient, it is impossible for callers to achieve this due to the difficulty of accomplishment both in technique and in cost. Fortunately, a 10–7.5% error rate enabled SV calling to be sufficiently sensitive and accurate; to be more specific, Sniffles and cuteSV obtained a 0.79 F1 score and 0.80 F1 score, respectively.

Next, we assessed the impact of the error rate on the four SV types. From the results shown in Additional file 1: Figure S3 and Table S6, we observed that the F1 scores of deletion were stable on data with diverse sequencing errors and obtained a 0.842 F1 score on average. In contrast, decreasing the sequencing error rate could significantly increase the performance in detecting insertions, and in regard to the 10–7.5% error rate, the F1 score was nearly 0.742. In particular, the average F1 score of discovering insertions surpassed 0.784 below 1% error rate data and made a great improvement compared with other datasets. Unfortunately, with the decreasing error rate, the F1 score of duplications and inversions decreased by 0.047 and 0.079 from 20 to 2.5% in error rates, respectively. It is a good choice to discover duplication and inversion precisely and sensitively on the data with less than 1% sequencing errors if the funds are sufficient. Nevertheless, the detection of inversions and duplications remains a bottleneck, and sequencing errors have a limited impact on their discovery.

We then evaluated the impact of the error rate on SVs under six size scales. The F1 scores of most size scales were satisfactory, i.e., 0.680, 0.779, 0.736, 0.787, 0.759, and 0.787 for the six scales (Additional file 1: Figures S3E to S3J and Table S7). To some extent, the detection for large-scale SV is easier than that for small-scale SV. It is worth noting that the detection of SV with 1–5 kbp performed best in the data with a 10–7.5% error rate among almost all datasets. In other words, a 10–7.5% error rate was sufficient to detect adequate SVs among almost all size scales. Moreover, higher performance was achieved when applying data with error rates below 1% for most size scales, especially for SVs shorter than 500 bp.

When considering genotypes, the average F1 score of the four SV callers also increased along with the reduced sequencing error rate. Notably, a 10–7.5% error rate was still a sufficient setting for SV calling; i.e., cuteSV surpassed 0.76 and Sniffles reached nearly 0.75 (Additional file 1: Table S7), and the datasets below a 1% error rate also had the ability to discover most SVs with high performance.

### Evaluation of the ensemble SV calling

With a combination and integration of multiple SV callsets, ensemble SV calling can improve the variant concordance of each SV caller. Here, we assessed the performance of ensemble SV calling performed by SURVIVOR [30] on the three sequencing attributes (i.e., coverage, read length, and error rate). It is evident from the ensemble calling method that the performance was promoting and generating more accurate and sensitive SV callsets, along with increasing coverage, read length and accuracy rate. Even when adopting the poor settings of the sequencing attributes, the ensemble calling method would still generate much better callsets than the single caller.

**Fig. 5:**
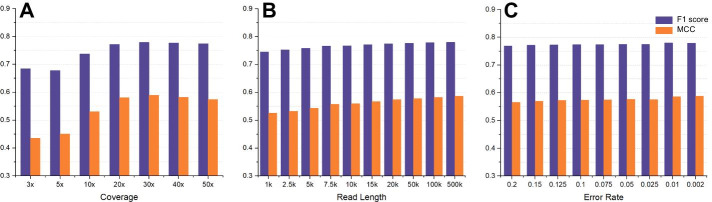
Comparison of the ensemble SV calling performance under diverse sequencing attributes. **a**–**c** Indicate the F1 scores and MCC rates of the ensemble SV calling method regarding coverage, read length and error rate, respectively

Figure 5a and Additional file 1: Table S8 show that coverage was still a vital attribute and that it enabled the ensemble method to achieve more sensitivity and accuracy under high coverage data. On the 20× dataset, the ensemble method obtained a 0.772 F1 score, indicating that 20× was still an appropriate coverage setting for discovering SVs with excellent resolution. In addition, the predicted trend curve shown in Additional file 1: Table S9 also supported the opinion that 36× coverage would enable the ensemble method to obtain the best SV results.

With the increase in read length, the F1 score increased steadily as the single callers did and had an average F1 score of approximately 0.766 from 1 to 500 kbp in read length (Fig. 5 and Additional file 1: Table S10). It is worth noting that the F1 score and MCC rate of ensemble calling surpassed 0.774 and 0.574 on the 20 kbp dataset, respectively. However, due to the relatively poor correlation coefficient of approximately 0.591 for the trend curve, it was difficult to give an appropriate prediction for the best read length.

In regard to the sequencing error rate, the average F1 scores for the ensemble approach were 0.774 and 0.780 on the datasets with 10–7.5% and below 1% error rates, respectively (Fig. 5 and Additional file 1: Table S11). The average MCC also maintained 0.574 and 0.586 on the data mentioned above. Unfortunately, the trend curve we fitted for ensemble calling was inconsistent with every single caller. Although the sequencing attributes of the recommendation could not reproduce the optimal result in ensemble calling, the performance achieved with the suggested settings still outperformed significantly.

### Evaluation of the joint effect of recommended settings

**Fig. 6:**
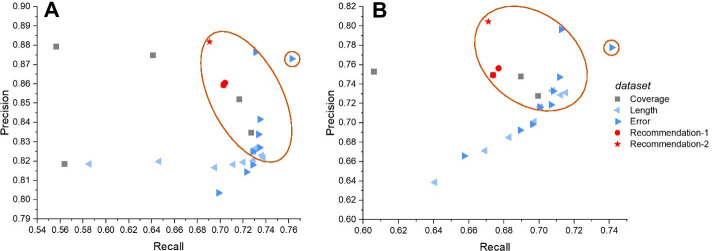
Comparison of the SV calling performance using the recommended datasets and 26 other datasets. Relationship between the recall and precision of variant calling tools on 29 datasets **a** without and **b** with genotyping. The Coverage, Length and Error legends indicate that the datasets vary in sequencing coverage, average read length and sequencing error rate, respectively. Recommendation-1 represents datasets with 10% and 7.5% error rates, 20× sequencing coverage and 20 kbp read length, and Recommendation-2 represents the dataset with a 1% error rate using the same coverage and read length. Datasets in the orange circular area have better performance

To illustrate that the recommended settings could also achieve satisfactory performance among all datasets mentioned above, we generated another three simulated datasets with the recommended settings, including 10%, 7.5% and 1% error rates, 20× sequencing coverage and 20 kbp average read length, to further determine their joint effect. From Fig. 6, datasets in the orange circular area show great potential to discover more SVs than others, and they all surpassed 0.77 and 0.70 F1 scores in detecting SVs without and with genotyping, which indicates a satisfactory performance in downstream analysis. The recommended datasets were also located in circular areas and had averages of 0.773 and 0.716 F1 scores, which outperformed many datasets (see more details in Additional file 1: Table S12). Although some datasets with higher coverage and longer read length performed the same as or slightly better than the datasets we recommend, we still suggest the optimal settings of long-read data with 10%, 7.5% and 1% error rates, 20× sequencing coverage and 20 kbp read length for a much lower cost and relatively better performance. In regard to the performance in detecting SVs on various types and size scales, the recommended datasets had an evident advantage on each set in terms of both the performance and cost (see more details in Additional file 1: Figure S5 and Table S12).

### Which is the most influential sequencing setting in SV calling?

**Fig. 7:**
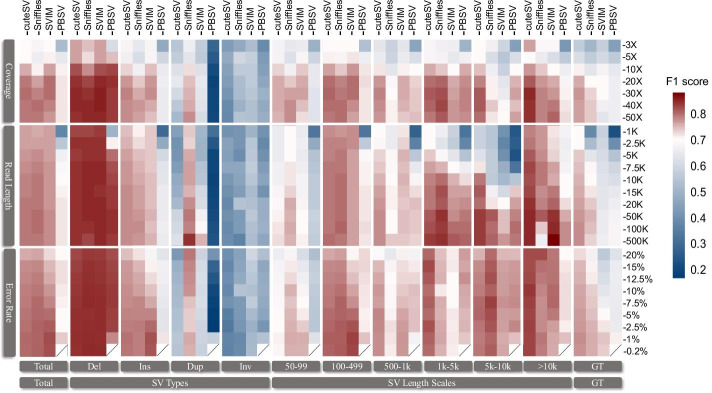
Heatmap of F1 scores for the SV detection performance with various sequencing metrics

To further determine which sequencing attributes (i.e., coverage, read length, and error rate) are the most influential factors that determine the performance regarding SV calling, we drew a heatmap of F1 scores under various sequencing settings and tools under all kinds of SV detection targets in this study. For the subplots labeled with “Total” and “SV Types” in Fig. 7, we found that coverage was crucial in SV calling since it achieved a significant advancement of F1 scores from low depth to high depth. In contrast, there was no noteworthy increase in F1 scores for read length and error rate under their various settings. For the subplots of the small SVs (less than 1 kbp), coverage was still the most influential factor, followed by read length and error rate. However, for the subplots of the middle-sized SVs (approximately 1–10 kbp), read length became a key point in obtaining higher F1 scores with the assistance of a longer average length of reads. In regard to genotypes (subplots labeled with “GT” in Fig. 7), read length could provide slightly more support to calculate accurate genotypes even if the growth of F1 scores under each sequencing setting was limited.

## Discussion

The rapid development of long-read sequencing technologies brings great opportunities for the detection of SV at an unprecedented resolution. However, there remains a challenge in balancing economic cost and performance. To break the bottlenecks, we generated a series of simulated long reads with different sequencing settings from a well-studied human sample and performed a comprehensive analysis to determine the most economical sequencing strategy on specific sequencing attributes. To achieve these, six state-of-the-art SV callers and one ensemble calling method were evaluated on 26 specifically designed simulated datasets. After comprehensively assessing F1 score, precision, recall, and MCC rate on a variety of SV types, sizes, zygosities and the joint effect of recommended settings, it was observed that the sequencing attributes with 20× coverage, 20 kbp average read length, and 10–7.5% or below 1% error rates enabled SV callers to obtain satisfactory performance and maintain low economic cost. Moreover, compared with read length and sequencing error, coverage might be the most influential factor affecting the performance of SV calling.

Recently, Genome in a Bottle Consortium (GIAB) created a robust benchmarking truth set including germline large deletions and insertions for the HG002 sample [31]. With these high-quality SV callsets, several SV callers achieved over 0.9 F1 scores. However, these SV callers hardly ever obtained similar results on the F1 score in this study, even for the data with abundant read coverage, length and negligible sequencing error. This was mainly because the entire genome region and all types of SV (apart from translocation) were taken into account for evaluation in our study, while only deletions and insertions in the genomic region within high confidence were included in the callsets of GIAB. Hence, it is more persuasive for this guideline of sequencing settings to rely on a comprehensive evaluation.

As an inexpensive and unbiased alternative, in silico simulations with the available ground truth have great potential for estimating the performance of SV calling. In addition, with the entrance of an era where genomic research has a large demand for large-scale sequencing datasets, in silico simulations have been the most effective tool for the simulation approach to handle various genomic problems. Although benchmarking with simulated reads is useful, it fails to reflect performance in real-world scenarios, especially for producing extremely complex mutations and for matching the characteristics of actual SV distribution.

The evaluation implemented in this work mainly concentrated on long-read alignment-based SV callers. While assembly-based SV discovery methods have large advantages regarding the pairwise comparison of genomes and enable SV calling even in the absence of a suitable reference genome, we excluded those approaches cause uncover SVs through assembly may require for unacceptable long-read sequencing coverage datasets, which is generated from other sequencing platforms.

In summary, long-read sequencing technologies promote SV discovery with ever-increasing accuracy, resolution and comprehensiveness. Due to the expensive costs and variable sequencing attributes, instructive suggestions for scientists and researchers are urgently needed. Our study aims to provide a practical guideline to balance economic costs and SV detection performance and to lead to more novel biological insights in routine research and clinical practice.

## Conclusions

Wide-development long-read sequencing technologies enable the discovery of full-spectrum SVs and reveal more novel biological insights. However, the expensive sequencing costs and variable sequencing attributes greatly limit SV calling based on long reads. We performed a comprehensive benchmark on a series of specifically designed simulated datasets with different sequencing coverages, read lengths, and error rates using state-of-the-art SV callers. The benchmark results demonstrate that satisfactory performance is achieved when the long-read data reach 20× coverage, 20 kbp average read length and approximately 10–7.5% or below 1% error rates. These recommended settings of long-read sequencing for SV detection will have extraordinary guiding significance in downstream research.

## Methods

### Generation of simulated datasets

First, we selected a well-studied human sample, CHM1 [2], as the baseline genome and extracted 19,607 SVs from these sample callsets under nstd162 and nstd137 [18] using dbVAR [32] as the ground truth. These SVs contained 6915 deletions, 12,250 insertions, 50 inversions, and 392 duplications, and all of them were over 50 bp in size and did not overlap with each other. VISOR [33], a standard sequencing read simulator, was used to integrate the SVs mentioned above into the reference genome and produced 26 synthetic diplontic long-read datasets (see Table 1) using various sequencing attributes (i.e., sequencing coverage, read length, and error rate). Note that we manually selected 50% autosomes for the generation of homozygous SVs, while the other autosomes and sex chromosomes were selected for heterozygous SVs, which were realized by setting different purities in VISOR input; e.g., 100.0 indicates a homozygous region and 50.0 indicates a heterozygous region. Afterward, Minimap2 [14] was used to complete alignment of simulated reads against the hs37d5 human reference genome [1], and SAMtools [34] was employed for the postprocessing of alignments, including sorting and indexing.

**Table 1:** Detailed settings of coverage, read length, and error rate on the 26 simulated long-read datasets

Adjusting variable	Value	Control variable
Coverage	3×, 5×, 10×, 20×, 30×, 40×, 50×	Read length = 20 kbp and error rate = 10%
Read length	1 k, 2.5 k, 5 k, 7.5 k, 10 k, 15 k, 20 k, 50 k, 100 k, 500 k	Coverage = 50× and error rate = 10%
Error rate	20%, 15%, 12.5%, 10%, 7.5%, 5%, 2.5%, 1%, 0.2%	Coverage = 50× and read length = 20 kbp

### Detection of structural variants

We applied six state-of-the-art long-read-based SV detection tools (see Table 2 and Additional file 1: Table S13) to complete SV calling on the 26 different simulated datasets with various sequencing settings. All tools were available to various types of SVs apart from NanoSV, which could not detect inversions from long-read alignments. In terms of the datasets with diverse sequencing depths, the number of the minimum supporting reads of each approach was adjusted to find more accurate SVs on each coverage. For more details about the implementation of SV callers, please see https://github.com/SQLiu-youyou/The-commands-of-the-evaluation.

**Table 2:** The SV calling methods used in this benchmark

Tool	Version	Availability
cuteSV	1.0.10	https://github.com/tjiangHIT/cuteSV
NanoSV	1.2.4	https://github.com/mroosmalen/nanosv
NanoVar	1.3.8	https://github.com/cytham/nanovar
PBSV	2.3.0	https://github.com/PacificBiosciences/pbsv
Sniffles	1.0.12	https://github.com/fritzsedlazeck/Sniffles
SVIM	1.4.0	https://github.com/eldariont/svim
SURVIVOR	1.0.7	https://github.com/fritzsedlazeck/SURVIVOR

In addition, we also implemented an ensemble-based approach for comprehensively discovering SVs from the callset of each SV caller. SURVIVOR, an SV combination and comparison toolkit, was applied to complete SV merging according to various scenarios, e.g., SV types, locations, number of supporting callers, etc. (see Table 2 and Additional file 1: Table S13). The ensemble-based approach only keeps those SV calls that can be detected by at least two SV callers within a close neighbor region that could be selected, while other SVs would probably be filtered out. This further assures the high performance of SV detection to infer the corresponding better attributes of sequencing reads.

### Evaluation of SV calling performance

To comprehensively benchmark the influence of sequencing settings on SV callings, Truvari (https://github.com/spiralgenetics/truvari) was used to assess the precision, recall, and F1 score of the callsets produced by various callers under diffent datasets. The details of these indicators are shown below.

Precision and recall are computed based on the TP (true positive) and the total number of SVs:

1 $$ precision = \frac{{TP_{p} }}{{total_{p} }} $$

2 $$ recall = \frac{{TP_{a} }}{{total_{a} }} $$

where $TP_{p}$ and $total_{p}$ indicate the number of true positive predictions and total predictions, and $TP_{a}$ and $total_{a}$. indicate the number of true positive answers and total answers, respectively.

Therefore, we can use the measurement of weighted averaging of both precision and recall, that is, the F1 score, to estimate the SV calling performance, which is defined as:

3 $$ F1 = \frac{2 \times precision \times recall}{{precision + recall}} $$

Apart from the indicators mentioned before, we also introduced the Matthews correlation coefficient to obtain a more reliable statistical rate among the true positives, false negatives, true negatives, and false positives, which is calculated as:

4 $$ MCC = \frac{TP*TN - FP*FN}{{\sqrt {\left( {TP + FP} \right)\left( {TP + FN} \right)\left( {TN + FP} \right)\left( {TN + FN} \right)} }} $$

where TP, TN, FP and FN indicate $$TP_{p}$$, $$TP_{a}$$, $$total_{p}$$- $$TP_{p} $$ and $$total_{a }$$- $$TP_{a}$$ calculated through Truvari, respectively. The value of MCC ranges from − 1 to + 1, where + 1 indicates a perfect SV detection model and − 1 indicates a poor model. A higher MCC score is achieved if and only if the prediction obtained good results in all four confusion matrix categories.

## Supplementary Information

**Additional file 1.** Supplementary Figures and Tables.

## Data Availability

All data generated or analyzed during this study are included in this published article and its additional information file. More detailed commands are available at https://github.com/SQLiu-youyou/The-commands-of-the-evaluation.
