# Supplementary material for: Long-read sequencing settings for efficient structural variation detection based on comprehensive evaluation
Source: BMC Bioinformatics. 2021 Nov 12;22:552. doi: 10.1186/s12859-021-04422-y (PMC8588741; doi:10.1186/s12859-021-04422-y)
Supplement: Supplementary file 1 — Additional file 1. Supplementary Figures and Tables. [file 12859_2021_4422_MOESM1_ESM.docx]

Title page

Long-read sequencing settings for efficient structural variation detection based on comprehensive evaluation

Tao Jiang^1, #^, Shiqi Liu^1, #^, Shuqi Cao^1^, Yadong Liu^1^, Zhe Cui^1^, Yadong Wang^1, *^ and Hongzhe Guo^1, *^

^1^Faculty of Computing, Harbin Institute of Technology, Harbin 150001, China.

^#^The authors wish it to be known that, in their opinion, the first two authors should be regarded as Joint First Authors.

^*^To whom correspondence should be addressed: [ydwang@hit.edu.cn](mailto:ydwang@hit.edu.cn) and [hzguo@hit.edu.cn](mailto:hzguo@hit.edu.cn).

Biographical Note:

**Tao Jiang** is a lecturer in Faculty of Computing at Harbin Institute of Technology. His work is focused on developing of structural variation discovering approach for long-read sequencing technologies.

**Shiqi Liu** is a graduate student in Faculty of Computing at Harbin Institute of Technology. She currently works for benchmarking on long-read sequencing technologies.

**Shuqi Cao** is a graduate student in Faculty of Computing at Harbin Institute of Technology. She currently works for large-scale population-based human genetic variants analysis.

**Yadong Liu** is a PhD student in Faculty of Computing at Harbin Institute of Technology. He works on the detection of SNPs/Indels and structural variation based on the alignment skeletons.

**Zhe Cui** is a PhD student in Faculty of Computing at Harbin Institute of Technology. He is working on the variant calling based on the Next Generation Sequencing.

**Yadong Wang** is a Professor in Faculty of Computing at Harbin Institute of Technology. His expertise is computational biology, knowledge engineering and machine learning.

**Hongzhe Guo** is a lecturer in Faculty of Computing at Harbin Institute of Technology. His work is focused on genome read alignment and variant calling based on the Next Generation sequencing and long-read sequencing technologies.

**Content**

[**Figure S1. Comparison of the SV calling performance under diverse sequencing coverages.** 4](#_Toc84006697)

[**Figure S2. Comparison of the SV calling performance under diverse average read lengths.** 6](#_Toc84006698)

[**Figure S3. Comparison of the SV calling performance under diverse error rates.** 8](#_Toc84006699)

[**Figure S4. Comparison of the ensemble SV calling performance under diverse sequencing attributes.** 9](#_Toc84006700)

[**Figure S5. Comparison of the SV calling performance using the recommended datasets and 26 other datasets.** 11](#_Toc84006701)

[**Table S1. The average F1 score, MCC rate, recall and precision of total performance under various sequencing coverages.** 12](#_Toc84006702)

[**Table S2. The F1 score, MCC and recall of total performance for SV calling under various coverages.** 13](#_Toc84006703)

[**Table S3. The estimated results of different sequencing attribute settings and their corresponding F1 scores under trend curve of each SV caller.** 15](#_Toc84006704)

[**Table S4. The average F1 score, MCC rate, recall and precision of total performance under various read lengths.** 16](#_Toc84006705)

[**Table S5. The F1 score and recall of total performance for SV calling under various sequencing read lengths.** 17](#_Toc84006706)

[**Table S6. The average F1 score, MCC rate, recall and precision of total performance under various error rates.** 19](#_Toc84006707)

[**Table S7. The F1 score and recall of total performance for SV calling under various sequencing error rates.** 20](#_Toc84006708)

[**Table S8. The F1 score, MCC rate, recall and precision of presence and F1 score of genotypes for ensemble calling under various coverages.** 21](#_Toc84006709)

[**Table S9. The estimated results of coverage, read length, error rate and F1 score under trend curve of ensemble calling.** 22](#_Toc84006710)

[**Table S10. The F1 score, MCC rate, recall and precision of presence and F1 score of genotypes for ensemble calling under various read lengths.** 23](#_Toc84006711)

[**Table S11. The F1 score, MCC rate, recall and precision of presence and F1 score of genotypes for ensemble calling under various error rates.** 24](#_Toc84006712)

[**Table S12. The average F1 score under different sizes and types, MCC rate, recall and precision using recommended datasets.** 25](#_Toc84006713)

[**Table S13. Detailed description of SV callers.** 26](#_Toc84006714)

**Figure S1. Comparison of the SV calling performance under diverse sequencing coverages.**

(**A**) to (**D**) represent F1 score of each SV caller on deletion, insertion, duplication and inversion calling, respectively. (**E**) to (**J**) represent F1 score of each SV caller for different sizes of SV calling, i.e., **(E)** 50~99 bp, **(F)** 100~499 bp, **(G)** 500~999 bp, **(H)** 1k~5k bp, **(I)** 5k~10k bp and **(J)** over 10k bp. Dark and light colors indicate the SV calling considering genotype and only presence. The red dotted line in each diagram indicates the overall trend with the sequencing depths.

**Figure S2. Comparison of the SV calling performance under diverse average read lengths.**

(**A**) to (**D**) represent F1 score of each SV caller on deletion, insertion, duplication and inversion calling, respectively. (**E**) to (**J**) represent F1 score of each SV caller for different sizes of SV calling, i.e., **(E)** 50~99 bp, **(F)** 100~499 bp, **(G)** 500~999 bp, **(H)** 1k~5k bp, **(I)** 5k~10k bp and **(J)** over 10k bp. Dark and light colors indicate the SV calling considering genotype and only presence. The red dotted line indicates the overall trend with the read lengths.

**Figure S3. Comparison of the SV calling performance under diverse error rates.**

(**A**) to (**D**) represent F1 score of each SV caller on deletion, insertion, duplication and inversion calling, respectively. (**E**) to (**J**) represent F1 score of each SV caller for different sizes of SV calling, i.e., **(E)** 50~99 bp, **(F)** 100~499 bp, **(G)** 500~999 bp, **(H)** 1k~5k bp, **(I)** 5k~10k bp and **(J)** over 10k bp. Dark and light colors indicate the SV calling considering genotype and only presence. The red dotted line indicates the overall trend with the sequencing error rates. It is worth noting that the average performance in 0.2% error data only consists of cuteSV, Sniffles and SVIM, and PBSV was excluded due to its relatively poor results.

**Figure S4. Comparison of the ensemble SV calling performance under diverse sequencing attributes.**

**(A)** to **(C)** indicate the recall and precision of the ensemble SV calling method regarding coverage, read length and error rate, respectively.

**Figure S5. Comparison of the SV calling performance using the recommended datasets and 26 other datasets.**

(**A**) to (**D**) represent relationship between recall and precision of 29 datasets on detecting deletion, insertion, duplication and inversion calling with/without genotyping, respectively. (**E**) to (**J**) represent relationship between recall and precision of 29 datasets on detecting different sizes of SV calling with/without genotyping, i.e., **(E)** 50~99 bp, **(F)** 100~499 bp, **(G)** 500~999 bp, **(H)** 1k~5k bp, **(I)** 5k~10k bp and **(J)** over 10k bp. The Coverage, Length and Error indicate the datasets vary in sequencing coverage, average read length and sequencing error rate, respectively. Recommendation-1 represents datasets with 10% and 7.5% error rates, 20× sequencing coverage and 20k bp read length, and Recommendation-2 represents the dataset with a 1% error rate using the same coverage and read length. Datasets in the orange circular area have better performance. The datasets in the orange circular area in detecting deletion, insertion, duplication and inversion surpassed 0.830, 0.730, 0.540 and 0.480 F1 score, respectively. For different size scales, they surpassed 0.680, 0.770, 0.740, 0.790, 0.710 and 0.760 F1 score, respectively.

**Table S1. The average F1 score, MCC rate, recall and precision of total performance under various sequencing coverages.**

|  | **3x** | **5x** | **10x** | **20x** | **30x** | **40x** | **50x** | |
| --- | --- | --- | --- | --- | --- | --- | --- | --- |
| **Average-F1** | | | | | | | | |
| **Total** | **45.15%** | 47.63% | 53.95% | 58.75% | 60.15% | 60.30% | **60.32%** | |
| **Deletion** | **50.05%** | 52.70% | 59.22% | 64.27% | 66.20% | 66.71% | **67.06%** |  |
| **Insertion** | **42.65%** | 45.03% | 51.36% | 56.04% | 57.46% | 57.56% | **57.48%** |  |
| **Duplication** | **36.81%** | 35.94% | 40.81% | 43.51% | 42.61% | 41.45% | **40.89%** |  |
| **Inversion** | **38.08%** | 42.47% | 41.37% | 40.73% | 40.47% | 38.64% | **39.70%** |  |
| **Total-GT** | 36.43% | 41.57% | 48.57% | 52.99% | 54.22% | 53.98% | 53.71% |  |
| **Average-MCC** | | | | | | | | |
| **Total** | **0.149** | 0.214 | 0.271 | 0.300 | 0.302 | 0.296 | **0.292** | |
| **Average-Recall** | | | | | | | | |
| **Total** | **38.35%** | 38.32% | 45.53% | 51.83% | 53.99% | 55.13% | **55.64%** | |
| **Average-Precision** | | | | | | | | |
| **Total** | 76.50% | **83.10%** | 81.57% | 78.15% | 76.22% | 74.45% | **73.55%** | |

**Note:** All the numbers in bold are mentioned in the main article.

**Table S2. The F1 score, MCC and recall of total performance for SV calling under various coverages.**

|  | | **3x** | **5x** | **10x** | **20x** | **30x** | **40x** | **50x** |
| --- | --- | --- | --- | --- | --- | --- | --- | --- |
| **F1** | | | | | | | | |
| **Average** | Total | 45.15% | 47.63% | 53.95% | 58.75% | 60.15% | 60.30% | 60.32% |
|  | 50-99 | 39.74% | 44.02% | 49.35% | 53.06% | 53.82% | 53.47% | 53.21% |
|  | 100-499 | 46.02% | 48.64% | 54.99% | 59.29% | 60.54% | 60.44% | 60.45% |
|  | 500-999 | 40.30% | 44.01% | 50.46% | 55.92% | 57.62% | 57.99% | 58.15% |
|  | 1k-5k | 43.28% | 46.47% | 53.20% | 59.11% | 61.01% | 61.48% | 60.99% |
|  | 5k-10k | 41.70% | 42.61% | 47.49% | 53.18% | 55.75% | 57.22% | 57.64% |
|  | >10k | 45.49% | 44.02% | 52.01% | 57.16% | 58.40% | 58.75% | 59.24% |
| **cuteSV** | Total | 70.65% | 70.23% | 76.06% | **79.95%** | 80.09% | 79.66% | 79.19% |
|  | 50-99 | 60.66% | 65.24% | 70.29% | 72.16% | 71.63% | 70.34% | 68.94% |
|  | 100-499 | 70.71% | 71.07% | 76.63% | 80.32% | 80.47% | 79.88% | 79.43% |
|  | 500-999 | 64.27% | 65.45% | 72.11% | 78.17% | 78.55% | 77.96% | 77.38% |
|  | 1k-5k | 68.09% | 68.65% | 75.84% | 81.26% | 81.22% | 80.77% | 79.39% |
|  | 5k-10k | 70.70% | 70.07% | 74.19% | 80.78% | 81.18% | 81.31% | 80.32% |
|  | >10k | 76.57% | 69.88% | 77.48% | 82.94% | 84.71% | 83.96% | 84.14% |
| **Sniffles** | Total | 68.43% | 66.01% | 71.15% | **76.94%** | 78.94% | 79.92% | 79.71% |
|  | 50-99 | 60.87% | 60.83% | 65.03% | 71.41% | 73.84% | 74.57% | 75.64% |
|  | 100-499 | 70.16% | 68.36% | 73.65% | 78.35% | 79.71% | 80.22% | 80.14% |
|  | 500-999 | 58.30% | 59.32% | 64.16% | 68.88% | 70.69% | 70.80% | 70.83% |
|  | 1k-5k | 67.26% | 67.37% | 73.77% | 80.63% | 82.11% | 83.17% | 81.23% |
|  | 5k-10k | 67.01% | 62.37% | 66.14% | 70.76% | 70.94% | 73.76% | 71.78% |
|  | >10k | 69.21% | 65.63% | 73.68% | 77.23% | 78.37% | 78.93% | 78.75% |
| **SVIM** | Total | 70.62% | 71.08% | 76.71% | **79.77%** | 80.04% | 78.69% | 77.50% |
|  | 50-99 | 62.50% | 67.21% | 72.51% | 73.53% | 73.32% | 70.73% | 68.38% |
|  | 100-499 | 70.56% | 71.97% | 77.55% | 79.84% | 79.89% | 77.93% | 76.54% |
|  | 500-999 | 64.35% | 65.30% | 70.78% | 75.62% | 76.08% | 75.29% | 73.96% |
|  | 1k-5k | 66.21% | 65.01% | 70.12% | 76.02% | 76.72% | 77.20% | 77.11% |
|  | 5k-10k | 59.08% | 55.11% | 58.70% | 64.29% | 65.34% | 68.77% | 69.86% |
|  | >10k | 68.92% | 61.81% | 70.56% | 76.03% | 77.33% | 79.38% | 80.61% |
| **PBSV** | Total | 52.31% | 64.84% | 71.39% | 71.96% | 71.64% | 71.55% | 71.68% |
|  | 50-99 | 48.38% | 57.32% | 61.51% | 60.11% | 57.85% | 57.34% | 56.88% |
|  | 100-499 | 53.93% | 66.07% | 71.98% | 72.18% | 71.93% | 71.83% | 71.94% |
|  | 500-999 | 46.10% | 62.27% | 70.51% | 73.23% | 74.10% | 74.18% | 74.74% |
|  | 1k-5k | 50.26% | 67.08% | 75.62% | 78.18% | 78.36% | 78.81% | 78.01% |
|  | 5k-10k | 44.91% | 61.89% | 70.45% | 75.22% | 76.61% | 74.62% | 76.33% |
|  | >10k | 43.00% | 56.37% | 65.94% | 68.32% | 70.99% | 71.88% | 72.55% |
| **MCC** | | | | | | | | |
| **cuteSV** | Total | 0.425 | 0.470 | 0.560 | **0.620** | 0.620 | 0.608 | 0.597 |
|  | 50-99 | 0.213 | 0.352 | 0.435 | 0.458 | 0.445 | 0.414 | 0.383 |
|  | 100-499 | 0.417 | 0.467 | 0.555 | 0.616 | 0.617 | 0.604 | 0.594 |
|  | 500-999 | 0.287 | 0.356 | 0.461 | 0.566 | 0.572 | 0.559 | 0.548 |
|  | 1k-5k | 0.377 | 0.430 | 0.537 | 0.628 | 0.625 | 0.615 | 0.588 |
|  | 5k-10k | 0.446 | 0.461 | 0.513 | 0.623 | 0.627 | 0.627 | 0.606 |
|  | >10k | 0.572 | 0.479 | 0.590 | 0.673 | 0.704 | 0.683 | 0.685 |
| **Sniffles** | Total | 0.434 | 0.432 | 0.502 | **0.578** | 0.606 | 0.618 | 0.617 |
|  | 50-99 | 0.238 | 0.333 | 0.389 | 0.469 | 0.497 | 0.502 | 0.526 |
|  | 100-499 | 0.460 | 0.453 | 0.531 | 0.594 | 0.612 | 0.618 | 0.618 |
|  | 500-999 | 0.182 | 0.216 | 0.295 | 0.379 | 0.414 | 0.416 | 0.417 |
|  | 1k-5k | 0.408 | 0.445 | 0.524 | 0.629 | 0.655 | 0.670 | 0.645 |
|  | 5k-10k | 0.432 | 0.398 | 0.450 | 0.483 | 0.489 | 0.518 | 0.511 |
|  | >10k | 0.427 | 0.446 | 0.544 | 0.585 | 0.599 | 0.600 | 0.613 |
| **SVIM** | Total | 0.416 | 0.463 | 0.551 | **0.597** | 0.601 | 0.575 | 0.555 |
|  | 50-99 | 0.254 | 0.362 | 0.454 | 0.472 | 0.471 | 0.432 | 0.399 |
|  | 100-499 | 0.411 | 0.460 | 0.556 | 0.598 | 0.601 | 0.570 | 0.550 |
|  | 500-999 | 0.289 | 0.347 | 0.429 | 0.513 | 0.522 | 0.513 | 0.492 |
|  | 1k-5k | 0.363 | 0.398 | 0.452 | 0.535 | 0.545 | 0.550 | 0.545 |
|  | 5k-10k | 0.335 | 0.317 | 0.330 | 0.379 | 0.381 | 0.430 | 0.445 |
|  | >10k | 0.511 | 0.432 | 0.532 | 0.599 | 0.617 | 0.646 | 0.664 |
| **Recall** | | | | | | | | |
| **cuteSV** | Total | 64.72% | 57.95% | 65.74% | **71.87%** | 72.38% | 72.62% | 72.62% |
| **Sniffles** | Total | 56.35% | 51.60% | 57.90% | **66.42%** | 69.87% | 71.94% | 71.34% |
| **SVIM** | Total | 67.21% | 60.83% | 69.41% | **77.59%** | 79.27% | 81.19% | 82.30% |
| **PBSV** | Total | 37.20% | 52.22% | 63.39% | 65.28% | 65.14% | 65.10% | 65.32% |
| **F1-GT** | | | | | | | | |
| **cuteSV** | Total | 57.09% | 62.29% | 70.87% | **76.12%** | 77.14% | 76.77% | 76.45% |
| **Sniffles** | Total | 50.75% | 57.48% | 64.33% | 70.44% | 72.99% | 73.65% | 73.62% |
| **SVIM** | Total | 57.94% | 60.96% | 67.64% | 69.86% | 69.51% | 66.75% | 64.27% |
| **PBSV** | Total | 44.89% | 56.85% | 64.94% | 66.26% | 66.09% | 66.05% | 66.01% |

**Note:** All the numbers in bold are mentioned in the main article.

**Table S3. The estimated results of different sequencing attribute settings and their corresponding F1 scores under trend curve of each SV caller.**

| **Tool** | **Fitting formula** | **Correlation coefficients (r^2^)** | **Maximum value**  **(F1/ setting)** | **Estimated value**  **(F1/ setting)** |
| --- | --- | --- | --- | --- |
| **Sequencing depth** | | | | |
| cuteSV | y=-1.07E^-4^x^2^+7.39E^-3^x+6.84E^-1^ | 0.9317 | 0.8107/34$\times$ | >0.75/11$\times$~58$\times$ |
| Sniffles | y=-9.27E^-5^x^2^+7.64E^-3^x+6.45E^-1^ | 0.9597 | 0.8027/41$\times$ | >0.75/17$\times$~65$\times$ |
| SVIM | y=-1.18E^-4^x^2^+7.48E^-3^x+6.88E^-1^ | 0.9261 | 0.8065/32$\times$ | >0.75/10$\times$~54$\times$ |
| PBSV | y=-1.65E^-4^x^2^+1.09E^-2^x+5.63E^-1^ | 0.6537 | --/-- | --/-- |
| NanoSV | y=-2.85E^-4^x^2^+2.24E^-2^x+1.08E^-2^ | 0.9825 | 0.4496/39$\times$ | --/-- |
| NanoVar | y=-3.58E^-5^x^2^+3.49E^-3^x+1.75E^-2^ | 0.9587 | 0.1027/49$\times$ | --/-- |
| **Mean read length** | | | | |
| cuteSV | y=-5.41E^-7^x^2^+3.10E^-4^x+7.77E^-1^ | 0.5762 | --/-- | --/-- |
| Sniffles | y=-6.84E^-7^x^2^+3.69E^-4^x+7.79E^-1^ | 0.4098 | --/-- | --/-- |
| SVIM | y=-2.74E^-7^x^2^+1.63E^-4^x+7.68E^-1^ | 0.7437 | --/-- | --/-- |
| PBSV | y=-3.54E^-6^x^2^+1.99E^-3^x+6.11E^-1^ | 0.2393 | --/-- | --/-- |
| **Sequencing error ratio** | | | | |
| cuteSV | y=-0.75x^2^+1.56x-3.67E^-3^ | 0.9796 | 0.8061/1.04 | >0.79/0.89~1.18 |
| Sniffles | y=-1.10E^-1^x^2^+2.22x-0.30 | 0.9934 | 0.8111/1.0 | >0.80/0.90~1.10 |
| SVIM | y=0.42x^2^-0.26x+0.67 | 0.9948 | --/-- | --/-- |
| PBSV | y=2.62 x^2^ -1.07xE^-2^+0.70 | 0.0363 | --/-- | --/-- |

**Table S4. The average F1 score, MCC rate, recall and precision of total performance under various read lengths.**

|  | **1k** | | **2.5k** | | **5k** | | **7.5k** | | **10k** | | **15k** | | **20k** | | **50k** | | **100k** | | **500k** | |
| --- | --- | --- | --- | --- | --- | --- | --- | --- | --- | --- | --- | --- | --- | --- | --- | --- | --- | --- | --- | --- |
| **Average-F1** | | | | | | | | | | | | | | | | | | | |  |
| **Total** | **66.56%** | 71.54% | | 74.75% | | 75.77% | | 76.32% | | 76.86% | | 77.02% | | 77.37% | | 77.40% | | **77.26%** | |  |
| **Deletion** | **75.01%** | 81.07% | | 83.21% | | 84.05% | | 84.27% | | 84.36% | | **84.43%** | | 84.49% | | 84.39% | | **83.90%** | |  |
| **Insertion** | **62.45%** | 67.29% | | 71.41% | | 72.42% | | 73.07% | | 73.75% | | **73.82%** | | 74.10% | | 74.15% | | **74.06%** | |  |
| **Duplication** | **50.45%** | 47.46% | | 47.22% | | 48.91% | | 49.68% | | 51.30% | | **53.00%** | | 56.98% | | 57.90% | | **61.64%** | |  |
| **Inversion** | **43.84%** | 45.11% | | 45.18% | | 48.87% | | 47.42% | | 44.57% | | 48.94% | | 47.29% | | 46.30% | | **44.37%** | |  |
| **Total-GT** | 47.40% | 55.80% | | 63.45% | | 66.45% | | 67.80% | | 69.28% | | 70.09% | | 71.32% | | 71.57% | | 71.31% | |  |
| **Average-MCC** | | | | | | | | | | | | | | | | | | | |  |
| **Total** | 0.404 | 0.466 | | 0.512 | | 0.530 | | 0.540 | | 0.549 | | **0.554** | | 0.559 | | 0.560 | | 0.558 | |  |
| **Average-Recall** | | | | | | | | | | | | | | | | | | | |  |
| **Total** | **58.53%** | 64.65% | | 69.51% | | 71.14% | | 72.01% | | 72.89% | | 72.89% | | 73.62% | | 73.78% | | **73.12%** | |  |
| **Average-Precision** | | | | | | | | | | | | | | | | | | | |  |
| **Total** | 81.86% | 81.99% | | 81.67% | | 81.83% | | 81.95% | | 82.04% | | 82.51% | | 82.31% | | 82.19% | | 82.69% | |  |

**Note:** All the numbers in bold are mentioned in the main article.

**Table S5. The F1 score and recall of total performance for SV calling under various sequencing read lengths.**

|  | | **1k** | **2.5k** | **5k** | **7.5k** | **10k** | **15k** | **20k** | **50k** | **100k** | **500k** |
| --- | --- | --- | --- | --- | --- | --- | --- | --- | --- | --- | --- |
| **F1** | | | | | | | | | | | |
| **Average** | Total | 66.56% | 71.54% | 74.75% | 75.77% | 76.32% | 76.86% | **77.02%** | 77.37% | 77.40% | 77.26% |
|  | 50-99 | **58.62%** | 62.45% | 64.20% | 65.01% | 65.67% | 66.50% | **67.46%** | 68.69% | 69.21% | **68.97%** |
|  | 100-499 | **66.74%** | 72.78% | 76.04% | 76.63% | 76.58% | 76.89% | **77.01%** | 77.12% | 77.23% | **77.06%** |
|  | 500-999 | **58.73%** | 61.17% | 68.41% | 70.69% | 72.18% | 73.62% | **74.23%** | 74.18% | 73.97% | **74.78%** |
|  | 1k-5k | **56.83%** | 57.08% | 65.64% | 71.98% | 76.47% | 78.59% | **78.93%** | 80.81% | 81.39% | **82.47%** |
|  | 5k-10k | **45.16%** | 44.72% | 46.30% | 52.58% | 60.55% | 70.67% | **74.57%** | 79.53% | 79.55% | **80.19%** |
|  | >10k | **73.63%** | 73.94% | 74.78% | 76.65% | 76.71% | 77.68% | **79.01%** | 81.56% | 80.38% | **79.12%** |
| **cuteSV** | Total | 76.32% | 76.94% | 77.57% | 78.33% | 78.58% | 78.88% | **79.19%** | 79.57% | 79.58% | 79.70% |
|  | 50-99 | 65.78% | 65.53% | 65.71% | 66.67% | 66.99% | 68.22% | 68.94% | 71.04% | 71.86% | 72.25% |
|  | 100-499 | 77.83% | 78.47% | 78.73% | 79.07% | 79.11% | 79.28% | 79.43% | 79.66% | 79.69% | 79.78% |
|  | 500-999 | 69.41% | 69.14% | 73.21% | 73.81% | 75.25% | 76.30% | 77.38% | 78.00% | 77.74% | 78.31% |
|  | 1k-5k | 69.40% | 69.67% | 74.14% | 76.86% | 78.35% | 79.25% | 79.39% | 81.19% | 81.80% | 83.25% |
|  | 5k-10k | 58.95% | 58.95% | 62.76% | 71.43% | 79.14% | 81.42% | 80.32% | 83.54% | 83.61% | 83.68% |
|  | >10k | 78.66% | 79.37% | 81.18% | 82.78% | 82.23% | 83.03% | 84.14% | 84.85% | 84.74% | 84.60% |
| **Sniffles** | Total | 75.69% | 76.86% | 78.05% | 78.61% | 79.27% | 79.69% | **79.71%** | 80.10% | 80.03% | 79.25% |
|  | 50-99 | 69.39% | 70.59% | 70.64% | 71.71% | 72.01% | 73.05% | 75.64% | 74.91% | 76.24% | 75.32% |
|  | 100-499 | 77.30% | 78.36% | 79.52% | 79.49% | 79.95% | 80.05% | 80.14% | 80.17% | 80.66% | 79.58% |
|  | 500-999 | 65.50% | 65.86% | 68.94% | 69.30% | 70.19% | 71.08% | 70.83% | 70.55% | 70.05% | 73.63% |
|  | 1k-5k | 66.04% | 63.32% | 69.27% | 74.94% | 79.97% | 82.03% | 81.23% | 84.55% | 83.69% | 84.34% |
|  | 5k-10k | 55.31% | 55.31% | 57.14% | 56.99% | 56.52% | 66.83% | 71.78% | 81.13% | 80.26% | 80.61% |
|  | >10k | 76.98% | 77.63% | 77.99% | 79.21% | 79.15% | 77.87% | 78.75% | 81.17% | 73.54% | 66.05% |
| **SVIM** | Total | 76.45% | 76.66% | 76.62% | 76.99% | 77.19% | 77.48% | **77.50%** | 77.89% | 77.88% | 78.15% |
|  | 50-99 | 65.85% | 65.95% | 65.88% | 66.45% | 67.39% | 67.88% | 68.38% | 70.06% | 70.55% | 71.07% |
|  | 100-499 | 78.04% | 77.87% | 76.81% | 76.78% | 76.35% | 76.38% | 76.54% | 76.61% | 76.42% | 76.85% |
|  | 500-999 | 72.07% | 73.06% | 75.44% | 74.75% | 74.96% | 74.82% | 73.96% | 74.42% | 74.25% | 73.90% |
|  | 1k-5k | 58.89% | 57.23% | 64.42% | 70.19% | 75.14% | 76.38% | 77.11% | 78.33% | 79.85% | 81.61% |
|  | 5k-10k | 41.09% | 37.97% | 40.99% | 48.68% | 51.41% | 60.82% | 69.86% | 74.60% | 74.60% | 77.14% |
|  | >10k | 71.45% | 72.25% | 72.95% | 76.66% | 75.80% | 79.12% | 80.61% | 83.95% | 85.19% | 88.02% |
| **PBSV** | Total | 37.78% | 55.73% | 66.75% | 69.15% | 70.26% | 71.39% | 71.68% | 71.90% | 72.11% | 71.93% |
|  | 50-99 | 33.48% | 47.71% | 54.56% | 55.22% | 56.28% | 56.85% | 56.88% | 58.75% | 58.20% | 57.22% |
|  | 100-499 | 33.77% | 56.44% | 69.09% | 71.19% | 70.93% | 71.84% | 71.94% | 72.05% | 72.14% | 72.05% |
|  | 500-999 | 27.93% | 36.64% | 56.06% | 64.92% | 68.31% | 72.26% | 74.74% | 73.75% | 73.86% | 73.29% |
|  | 1k-5k | 32.98% | 38.10% | 54.72% | 65.93% | 72.44% | 76.70% | 78.01% | 79.17% | 80.21% | 80.67% |
|  | 5k-10k | 25.30% | 26.67% | 24.32% | 33.24% | 55.14% | 73.61% | 76.33% | 78.84% | 79.75% | 79.32% |
|  | >10k | 67.42% | 66.49% | 67.01% | 67.93% | 69.64% | 70.71% | 72.55% | 76.26% | 78.04% | 77.81% |
| **Recall** | | | | | | | | | | | |
| **cuteSV** | Total | 69.43% | 71.22% | 71.83% | 72.28% | 72.37% | 72.47% | **72.62%** | 72.52% | 72.50% | 72.12% |
|  | 50-99 | 67.30% | 68.95% | 66.86% | 66.56% | 65.66% | 65.52% | 65.41% | 64.91% | 64.76% | 64.18% |
|  | 100-499 | 73.06% | 75.27% | 75.74% | 75.66% | 75.42% | 75.21% | 75.07% | 74.71% | 74.60% | 74.32% |
|  | 500-999 | 64.67% | 66.06% | 71.90% | 73.43% | 75.55% | 77.30% | 78.03% | 78.39% | 78.39% | 78.25% |
|  | 1k-5k | 61.69% | 63.07% | 71.52% | 76.55% | 79.46% | 80.41% | 81.06% | 82.52% | 83.32% | 83.61% |
|  | 5k-10k | 45.34% | 45.34% | 49.80% | 62.75% | 74.49% | 78.95% | 80.16% | 82.19% | 82.59% | 80.97% |
|  | >10k | 71.15% | 71.37% | 74.40% | 76.14% | 76.79% | 78.52% | 81.13% | 85.03% | 86.12% | 84.60% |
| **Sniffles** | Total | 65.16% | 67.54% | 69.61% | 70.49% | 71.43% | 72.27% | **71.34%** | 73.09% | 73.46% | 71.77% |
|  | 50-99 | 65.39% | 68.80% | 69.47% | 69.90% | 69.49% | 69.68% | 69.17% | 69.43% | 69.86% | 67.49% |
|  | 100-499 | 67.82% | 70.33% | 72.32% | 72.40% | 73.21% | 73.66% | 73.00% | 73.80% | 74.44% | 72.50% |
|  | 500-999 | 60.29% | 61.39% | 67.01% | 68.54% | 69.78% | 71.68% | 69.56% | 72.12% | 72.04% | 71.24% |
|  | 1k-5k | 51.57% | 48.73% | 56.30% | 64.46% | 72.25% | 77.13% | 73.12% | 81.72% | 81.65% | 80.41% |
|  | 5k-10k | 40.08% | 40.08% | 41.30% | 42.11% | 42.11% | 54.25% | 58.70% | 75.71% | 75.71% | 74.90% |
|  | >10k | 68.55% | 71.15% | 74.19% | 73.97% | 72.89% | 71.37% | 68.33% | 78.52% | 80.48% | 84.82% |
| **SVIM** | Total | 74.12% | 76.62% | 78.84% | 80.30% | 81.06% | 81.93% | **82.30%** | 82.92% | 83.11% | 82.58% |
|  | 550-99 | 77.94% | 81.19% | 80.93% | 81.24% | 81.04% | 80.53% | 80.31% | 80.22% | 79.89% | 78.93% |
|  | 100-499 | 78.93% | 81.90% | 83.98% | 84.93% | 85.37% | 85.88% | 86.26% | 86.56% | 86.70% | 85.91% |
|  | 500-999 | 64.31% | 66.13% | 74.45% | 77.59% | 79.20% | 81.90% | 81.46% | 83.65% | 84.38% | 83.72% |
|  | 1k-5k | 45.23% | 43.26% | 52.29% | 60.89% | 68.24% | 72.54% | 73.85% | 76.98% | 79.10% | 80.63% |
|  | 5k-10k | 27.53% | 24.29% | 26.72% | 33.60% | 36.84% | 47.77% | 59.11% | 65.99% | 65.99% | 67.61% |
|  | >10k | 56.18% | 57.05% | 57.92% | 62.69% | 61.82% | 66.59% | 68.55% | 73.75% | 76.14% | 80.48% |
| **F1-GT** | | | | | | | | | | | |
| **cuteSV** | Total | 69.35% | 70.71% | 73.53% | 74.67% | 75.07% | 75.88% | **76.45%** | 77.38% | 77.74% | 78.21% |
| **Sniffles** | Total | 38.68% | 52.60% | 64.15% | 68.74% | 70.56% | 72.05% | 73.62% | 75.13% | 74.87% | 72.90% |
| **SVIM** | Total | 58.29% | 59.81% | 61.50% | 62.63% | 63.34% | 64.18% | 64.27% | 65.38% | 65.48% | 66.11% |
| **PBSV** | Total | 23.26% | 40.09% | 54.63% | 59.74% | 62.22% | 65.02% | 66.01% | 67.41% | 68.20% | 68.04% |

**Note:** All the numbers in bold are mentioned in the main article.

**Table S6. The average F1 score, MCC rate, recall and precision of total performance under various error rates.**

|  | **20%** | **15%** | **12.5%** | **10%** | **7.5%** | **5%** | **2.5%** | **1%** | **0.2%** |
| --- | --- | --- | --- | --- | --- | --- | --- | --- | --- |
| **Average-F1** | | | | | | | | | |
| **Total** | **74.01%** | 76.07% | 76.60% | 77.02% | 77.52% | 77.86% | 78.32% | 79.57% | **81.31%** |
| **Deletion** | **83.35%** | **83.88%** | **84.12%** | **84.43%** | **84.58%** | **84.46%** | **84.46%** | **83.24%** | **84.85%** |
| **Insertion** | 69.84% | 72.71% | 73.35% | **73.82%** | **74.52%** | 75.12% | 75.87% | **78.38%** | 80.31% |
| **Duplication** | **55.81%** | 54.37% | 54.00% | 53.00% | 52.12% | 51.66% | **51.15%** | 62.35% | 63.03% |
| **Inversion** | **49.90%** | 47.11% | 47.88% | 48.94% | 47.17% | 44.32% | **42.04%** | 45.57% | 42.82% |
| **Total-GT** | 64.95% | 68.13% | 68.88% | 70.09% | 70.70% | 71.65% | 72.64% | 74.96% | 75.72% |
| **Average-MCC** | | | | | | | | | |
| **Total-MCC** | 0.502 | 0.537 | 0.546 | 0.554 | 0.561 | 0.567 | 0.576 | 0.607 | 0.636 |
| **Average-Recall** | | | | | | | | | |
| **Total-Recall** | 69.85% | 72.29% | 72.83% | 72.89% | 73.44% | 73.33% | 73.45% | 73.10% | 76.25% |
| **Average-Precision** | | | | | | | | | |
| **Total-Precision** | **80.36%** | 81.44% | 81.81% | 82.51% | 82.71% | 83.39% | 84.16% | **87.64%** | **87.31%** |

**Note:** All the numbers in bold are mentioned in the main article.

**Table S7. The F1 score and recall of total performance for SV calling under various sequencing error rates.**

|  | | **20%** | **15%** | **12.5%** | **10%** | **7.5%** | **5%** | **2.5%** | **1%** | **0.2%** |
| --- | --- | --- | --- | --- | --- | --- | --- | --- | --- | --- |
| **F1** | | | | | | | | | | |
| **Average** | Total | 74.01% | 76.07% | 76.60% | 77.02% | 77.52% | 77.86% | 78.32% | 79.57% | 81.31% |
|  | 50-99 | 62.52% | 65.56% | 66.49% | 67.46% | 67.37% | 67.95% | 68.81% | 71.55% | 74.08% |
|  | 100-499 | 74.31% | 75.92% | 76.35% | 77.01% | 77.64% | 78.29% | 79.00% | 80.19% | 81.98% |
|  | 500-999 | 68.4% | 72.0% | 73.2% | 74.2% | 74.3% | 75.2% | 75.7% | 73.8% | 75.68% |
|  | 1k-5k | 76.57% | 78.46% | 78.92% | 78.93% | 80.10% | 79.42% | 79.34% | 77.74% | 79.21% |
|  | 5k-10k | 76.01% | 76.56% | 76.45% | 74.57% | 75.86% | 75.75% | 76.36% | 75.89% | 75.84% |
|  | >10k | 78.94% | 78.65% | 78.71% | 79.01% | 78.74% | 78.37% | 77.53% | 78.48% | 79.58% |
| **cuteSV** | Total | 76.17% | 78.29% | 78.64% | **79.19%** | **79.41%** | 79.92% | 80.14% | 80.71% | 80.48% |
|  | 50-99 | 66.28% | 67.98% | 68.34% | 68.94% | 69.21% | 69.60% | 70.25% | 71.23% | 70.91% |
|  | 100-499 | 76.37% | 78.43% | 78.97% | 79.43% | 79.64% | 80.39% | 80.77% | 81.42% | 81.11% |
|  | 500-999 | 69.42% | 75.10% | 76.29% | 77.38% | 76.91% | 78.58% | 78.48% | 78.08% | 78.07% |
|  | 1k-5k | 75.54% | 78.28% | 78.86% | 79.39% | 80.67% | 80.89% | 79.83% | 79.41% | 79.79% |
|  | 5k-10k | 80.72% | 82.82% | 81.71% | 80.32% | 81.36% | 80.48% | 82.26% | 81.21% | 80.48% |
|  | >10k | 82.71% | 82.63% | 82.98% | 84.14% | 83.58% | 83.11% | 83.46% | 83.28% | 83.26% |
| **Sniffles** | Total | 76.53% | 78.54% | 79.27% | **79.71%** | **80.67%** | 80.70% | 81.12% | 81.04% | 81.09% |
|  | 50-99 | 68.47% | 71.69% | 72.79% | 75.64% | 74.37% | 75.17% | 75.71% | 76.07% | 75.72% |
|  | 100-499 | 76.98% | 78.67% | 79.29% | 80.14% | 81.11% | 81.06% | 81.61% | 81.42% | 81.69% |
|  | 500-999 | 65.07% | 68.57% | 70.27% | 70.83% | 70.87% | 71.10% | 72.46% | 71.99% | 71.52% |
|  | 1k-5k | 80.49% | 81.65% | 82.38% | 81.23% | 83.54% | 82.50% | 82.07% | 80.91% | 81.43% |
|  | 5k-10k | 74.65% | 75.29% | 75.58% | 71.78% | 75.74% | 75.63% | 76.23% | 76.43% | 75.63% |
|  | >10k | 82.77% | 80.18% | 79.48% | 78.75% | 77.99% | 78.20% | 78.30% | 79.73% | 79.32% |
| **SVIM** | Total | 72.86% | 75.08% | 76.15% | 77.50% | 78.76% | 80.36% | 81.60% | 82.58% | 82.36% |
|  | 50-99 | 61.94% | 65.02% | 66.58% | 68.38% | 69.87% | 71.64% | 73.85% | 75.84% | 75.60% |
|  | 100-499 | 71.01% | 73.50% | 74.70% | 76.54% | 77.99% | 80.46% | 82.21% | 83.45% | 83.13% |
|  | 500-999 | 71.32% | 72.53% | 73.03% | 73.96% | 74.88% | 76.47% | 77.13% | 77.57% | 77.47% |
|  | 1k-5k | 75.22% | 76.69% | 77.01% | 77.11% | 77.82% | 77.04% | 76.89% | 76.06% | 76.41% |
|  | 5k-10k | 71.60% | 71.70% | 70.56% | 69.86% | 70.31% | 71.73% | 71.56% | 71.73% | 71.43% |
|  | >10k | 79.74% | 79.90% | 79.95% | 80.61% | 80.81% | 79.59% | 76.96% | 76.32% | 76.15% |
| **F1-GT** | | | | | | | | | | |
| **cuteSV** | Total | 72.78% | 75.20% | 75.72% | **76.45%** | **76.63%** | 77.11% | 77.38% | 78.14% | 77.66% |
| **Sniffles** | Total | 67.30% | 71.17% | 71.63% | **73.62%** | **74.02%** | 74.61% | 75.49% | 75.93% | 75.81% |
| **SVIM** | Total | 56.31% | 60.23% | 62.02% | 64.27% | 66.41% | 69.51% | 71.96% | 74.35% | 73.69% |
| **PBSV** | Total | 63.39% | 65.91% | 66.17% | 66.01% | 65.74% | 65.37% | 65.75% | 71.41% | - |

**Note:** All the numbers in bold are mentioned in the main article.

**Table S8. The F1 score, MCC rate, recall and precision of presence and F1 score of genotypes for ensemble calling under various coverages.**

|  | | **3x** | **5x** | **10x** | **20x** | **30x** | **40x** | **50x** |
| --- | --- | --- | --- | --- | --- | --- | --- | --- |
| **F1** | Total | 68.41% | 67.73% | 73.67% | **77.24%** | 77.84% | 77.68% | 77.37% |
|  | 50-99 | 61.09% | 62.60% | 67.65% | 70.14% | 70.28% | 69.63% | 69.23% |
|  | 100-499 | 69.35% | 68.96% | 74.70% | 77.97% | 78.38% | 77.89% | 77.33% |
|  | 500-999 | 64.92% | 65.15% | 71.85% | 76.20% | 77.32% | 78.22% | 77.47% |
|  | 1k-5k | 69.15% | 69.37% | 76.78% | 82.07% | 83.19% | 83.42% | 83.50% |
|  | 5k-10k | 68.69% | 69.19% | 74.52% | 80.91% | 80.63% | 82.20% | 82.43% |
|  | >10k | 70.79% | 64.03% | 72.37% | 76.61% | 77.65% | 78.43% | 79.27% |
| **MCC** | Total | 0.435 | 0.450 | 0.531 | 0.580 | 0.588 | 0.581 | 0.574 |
|  | 50-99 | 0.270 | 0.354 | 0.421 | 0.448 | 0.445 | 0.426 | 0.416 |
|  | 100-499 | 0.430 | 0.445 | 0.529 | 0.578 | 0.583 | 0.572 | 0.560 |
|  | 500-999 | 0.317 | 0.357 | 0.457 | 0.528 | 0.548 | 0.565 | 0.549 |
|  | 1k-5k | 0.427 | 0.460 | 0.564 | 0.648 | 0.668 | 0.670 | 0.671 |
|  | 5k-10k | 0.463 | 0.474 | 0.545 | 0.643 | 0.633 | 0.656 | 0.657 |
|  | >10k | 0.503 | 0.408 | 0.516 | 0.575 | 0.589 | 0.596 | 0.608 |
| **Recall** | Total | 56.25% | 53.89% | 61.71% | 67.22% | 68.28% | 68.58% | 68.54% |
|  | 50-99 | 51.11% | 49.12% | 55.53% | 59.63% | 60.33% | 60.38% | 60.31% |
|  | 100-499 | 59.06% | 56.83% | 64.93% | 70.30% | 71.28% | 71.23% | 70.85% |
|  | 500-999 | 58.03% | 54.31% | 64.38% | 72.55% | 74.67% | 76.93% | 76.79% |
|  | 1k-5k | 58.85% | 56.74% | 67.66% | 77.35% | 79.32% | 80.99% | 81.28% |
|  | 5k-10k | 55.06% | 55.47% | 62.75% | 72.06% | 72.47% | 75.71% | 76.92% |
|  | >10k | 57.05% | 49.24% | 59.65% | 65.73% | 67.46% | 69.41% | 70.93% |
| **Precision** | Total | 87.27% | 91.12% | 91.37% | 90.76% | 90.52% | 89.57% | 88.81% |
|  | 50-99 | 75.92% | 86.27% | 86.54% | 85.15% | 84.15% | 82.22% | 81.25% |
|  | 100-499 | 83.99% | 87.65% | 87.92% | 87.53% | 87.04% | 85.92% | 85.11% |
|  | 500-999 | 73.68% | 81.40% | 81.29% | 80.23% | 80.17% | 79.55% | 78.16% |
|  | 1k-5k | 83.82% | 89.23% | 88.73% | 87.41% | 87.47% | 86.00% | 85.85% |
|  | 5k-10k | 91.28% | 91.95% | 91.72% | 92.23% | 90.86% | 89.90% | 88.79% |
|  | >10k | 93.26% | 91.53% | 91.97% | 91.82% | 91.47% | 90.14% | 89.84% |

**Note:** All the numbers in bold are mentioned in the main article.

**Table S9. The estimated results of coverage, read length, error rate and F1 score under trend curve of ensemble calling.**

| **Parameter** | **Fitting formula** | **Correlation coefficients (r^2^)** | **Maximum value**  **(F1/setting)** | **Estimated value**  **(F1/setting)** |
| --- | --- | --- | --- | --- |
| coverage | y=-1.01E^-4^x^2^+7.17E^-3^x+6.61E^-1^ | 0.9384 | 0.7881/36$\times$ | >0.75/1$6\times$~55$\times$ |
| read length | y=-5.23E^-7^x^2^+3.02E^-4^x+7.59E^-1^ | 0.5908 | --/-- | --/-- |
| error rate | y=5.98E^-2^x^2^-5.70E^-2^x+7.76E^-1^ | 0.9242 | --/-- | --/-- |

**Table S10. The F1 score, MCC rate, recall and precision of presence and F1 score of genotypes for ensemble calling under various read lengths.**

|  | | **1k** | **2.5k** | **5k** | **7.5k** | **10k** | **15k** | **20k** | **50k** | **100k** | **500k** |
| --- | --- | --- | --- | --- | --- | --- | --- | --- | --- | --- | --- |
| **F1** | Total | 74.50% | 75.19% | 75.79% | 76.54% | 76.67% | 77.06% | **77.37%** | 77.56% | 77.78% | 77.92% |
|  | 50-99 | 66.74% | 66.91% | 66.73% | 67.01% | 67.58% | 68.46% | 69.23% | 70.32% | 71.25% | 71.54% |
|  | 100-499 | 76.53% | 77.41% | 77.04% | 77.42% | 76.96% | 77.00% | 77.33% | 77.65% | 77.78% | 77.88% |
|  | 500-999 | 70.39% | 71.20% | 74.59% | 75.90% | 75.65% | 77.48% | 77.47% | 76.67% | 77.82% | 77.35% |
|  | 1k-5k | 66.93% | 65.07% | 72.99% | 78.50% | 81.41% | 82.39% | 83.50% | 83.39% | 83.84% | 84.57% |
|  | 5k-10k | 55.10% | 55.34% | 56.28% | 59.69% | 71.26% | 80.71% | 82.43% | 83.90% | 83.30% | 84.48% |
|  | >10k | 75.71% | 76.49% | 78.76% | 78.57% | 78.27% | 76.89% | 79.27% | 81.59% | 84.28% | 86.25% |
| **MCC** | Total | 0.525 | 0.532 | 0.542 | 0.557 | 0.559 | 0.566 | **0.574** | 0.577 | 0.581 | 0.585 |
|  | 50-99 | 0.348 | 0.347 | 0.346 | 0.354 | 0.370 | 0.393 | 0.416 | 0.447 | 0.474 | 0.489 |
|  | 100-499 | 0.547 | 0.559 | 0.551 | 0.560 | 0.551 | 0.552 | 0.560 | 0.567 | 0.570 | 0.573 |
|  | 500-999 | 0.418 | 0.430 | 0.493 | 0.518 | 0.513 | 0.550 | 0.549 | 0.533 | 0.557 | 0.547 |
|  | 1k-5k | 0.398 | 0.355 | 0.484 | 0.579 | 0.631 | 0.649 | 0.671 | 0.668 | 0.677 | 0.692 |
|  | 5k-10k | 0.267 | 0.265 | 0.283 | 0.306 | 0.469 | 0.629 | 0.657 | 0.682 | 0.669 | 0.697 |
|  | >10k | 0.543 | 0.553 | 0.591 | 0.587 | 0.584 | 0.555 | 0.608 | 0.641 | 0.695 | 0.725 |
| **Recall** | Total | 64.67% | 66.20% | 67.02% | 67.79% | 68.02% | 68.40% | 68.54% | 68.79% | 69.03% | 68.93% |
|  | 50-99 | 60.70% | 62.02% | 61.03% | 60.86% | 60.56% | 60.52% | 60.31% | 60.25% | 60.23% | 59.71% |
|  | 100-499 | 69.41% | 71.49% | 71.26% | 71.33% | 70.77% | 70.70% | 70.85% | 70.86% | 71.07% | 70.97% |
|  | 500-999 | 64.82% | 66.86% | 72.63% | 75.04% | 75.40% | 77.96% | 76.79% | 77.01% | 78.61% | 77.52% |
|  | 1k-5k | 55.50% | 54.26% | 64.75% | 73.12% | 78.15% | 80.26% | 81.28% | 83.18% | 83.69% | 83.83% |
|  | 5k-10k | 40.49% | 40.89% | 41.70% | 46.15% | 60.73% | 73.68% | 76.92% | 80.16% | 79.76% | 79.35% |
|  | >10k | 66.59% | 68.11% | 71.58% | 71.58% | 70.72% | 69.63% | 70.93% | 75.92% | 78.52% | 85.03% |
| **Precision** | Total | 87.86% | 87.00% | 87.21% | 87.88% | 87.85% | 88.23% | 88.81% | 88.91% | 89.08% | 89.60% |
|  | 50-99 | 74.12% | 72.63% | 73.61% | 74.55% | 76.43% | 78.79% | 81.25% | 84.44% | 87.20% | 89.20% |
|  | 100-499 | 85.28% | 84.40% | 83.84% | 84.64% | 84.32% | 84.55% | 85.11% | 85.86% | 85.89% | 86.29% |
|  | 500-999 | 77.02% | 76.14% | 76.66% | 76.77% | 75.90% | 77.00% | 78.16% | 76.34% | 77.04% | 77.18% |
|  | 1k-5k | 84.29% | 81.24% | 83.63% | 84.73% | 84.96% | 84.64% | 85.85% | 83.60% | 83.99% | 85.32% |
|  | 5k-10k | 86.21% | 85.59% | 86.55% | 84.44% | 86.21% | 89.22% | 88.79% | 88.00% | 87.17% | 90.32% |
|  | >10k | 87.71% | 87.22% | 87.53% | 87.07% | 87.63% | 85.83% | 89.84% | 88.16% | 90.95% | 87.50% |

**Note:** All the numbers in bold are mentioned in the main article.

**Table S11. The F1 score, MCC rate, recall and precision of presence and F1 score of genotypes for ensemble calling under various error rates.**

|  | | **20%** | **15%** | **12.5%** | **10%** | **7.5%** | **5%** | **2.5%** | **1%** | **0.2%** |
| --- | --- | --- | --- | --- | --- | --- | --- | --- | --- | --- |
| **F1** | Total | 76.84% | 77.15% | 77.28% | **77.37%** | **77.44%** | 77.55% | 77.55% | **78.00%** | 77.93% |
|  | 50-99 | 67.59% | 68.29% | 68.70% | 69.23% | 68.98% | 68.85% | 69.01% | 69.33% | 69.89% |
|  | 100-499 | 77.19% | 77.12% | 77.36% | 77.33% | 77.48% | 77.97% | 78.15% | 78.63% | 78.79% |
|  | 500-999 | 74.77% | 76.52% | 76.04% | 77.47% | 77.51% | 77.33% | 77.15% | 77.13% | 76.03% |
|  | 1k-5k | 80.62% | 82.18% | 83.10% | 83.50% | 83.30% | 82.88% | 82.30% | 81.90% | 81.53% |
|  | 5k-10k | 82.48% | 82.76% | 83.01% | 82.43% | 82.66% | 82.94% | 82.91% | 82.33% | 78.98% |
|  | >10k | 82.24% | 80.81% | 79.04% | 79.27% | 78.19% | 78.26% | 78.19% | 80.23% | 81.70% |
| **MCC** | Total | 0.565 | 0.570 | 0.572 | **0.574** | **0.574** | 0.576 | 0.575 | **0.586** | 0.587 |
|  | 50-99 | 0.375 | 0.392 | 0.402 | 0.416 | 0.407 | 0.405 | 0.408 | 0.414 | 0.427 |
|  | 100-499 | 0.556 | 0.555 | 0.560 | 0.560 | 0.563 | 0.573 | 0.577 | 0.589 | 0.593 |
|  | 500-999 | 0.496 | 0.530 | 0.521 | 0.549 | 0.550 | 0.547 | 0.543 | 0.543 | 0.522 |
|  | 1k-5k | 0.618 | 0.645 | 0.664 | 0.671 | 0.666 | 0.658 | 0.646 | 0.638 | 0.633 |
|  | 5k-10k | 0.655 | 0.662 | 0.667 | 0.657 | 0.659 | 0.666 | 0.663 | 0.653 | 0.612 |
|  | >10k | 0.655 | 0.632 | 0.600 | 0.608 | 0.579 | 0.586 | 0.579 | 0.613 | 0.657 |
| **Recall** | Total | 67.78% | 68.26% | 68.41% | 68.54% | 68.73% | 68.85% | 68.98% | 69.17% | 68.69% |
|  | 50-99 | 59.86% | 59.99% | 60.26% | 60.31% | 60.45% | 60.33% | 60.56% | 60.83% | 61.11% |
|  | 100-499 | 70.82% | 70.71% | 70.82% | 70.85% | 70.86% | 71.24% | 71.32% | 71.37% | 71.42% |
|  | 500-999 | 73.21% | 76.50% | 76.20% | 76.79% | 77.74% | 77.30% | 77.01% | 76.42% | 74.09% |
|  | 1k-5k | 76.18% | 79.75% | 80.55% | 81.28% | 82.81% | 82.67% | 82.45% | 81.57% | 78.59% |
|  | 5k-10k | 78.14% | 77.73% | 78.14% | 76.92% | 78.14% | 77.73% | 78.54% | 77.33% | 69.23% |
|  | >10k | 76.36% | 73.54% | 71.15% | 70.93% | 71.15% | 70.28% | 71.15% | 74.84% | 73.10% |
| **Precision** | Total | 88.71% | 88.69% | 88.80% | 88.81% | 88.67% | 88.76% | 88.55% | 89.41% | 90.04% |
|  | 50-99 | 77.60% | 79.25% | 79.90% | 81.25% | 80.30% | 80.18% | 80.20% | 80.59% | 81.63% |
|  | 100-499 | 84.81% | 84.82% | 85.22% | 85.11% | 85.45% | 86.10% | 86.42% | 87.53% | 87.86% |
|  | 500-999 | 76.39% | 76.55% | 75.87% | 78.16% | 77.29% | 77.36% | 77.29% | 77.84% | 78.08% |
|  | 1k-5k | 85.60% | 84.75% | 85.80% | 85.85% | 83.79% | 83.09% | 82.15% | 82.23% | 84.69% |
|  | 5k-10k | 87.33% | 88.48% | 88.53% | 88.79% | 87.73% | 88.89% | 87.78% | 88.02% | 91.94% |
|  | >10k | 89.11% | 89.68% | 88.89% | 89.84% | 86.77% | 88.28% | 86.77% | 86.47% | 92.58% |

**Note:** All the numbers in bold are mentioned in the main article.

**Table S12. The average F1 score under different sizes and types, MCC rate, recall and precision using recommended datasets.**

|  | **10%** | **7.5%** | **1%** | **average** |
| --- | --- | --- | --- | --- |
| **Average-F1** | | | | |
| **Total** | 77.16% | 77.31% | 77.37% | **77.28%** |
| **50-99** | 69.30% | 69.72% | 69.59% | 69.54% |
| **100-499** | 77.67% | 77.89% | 78.43% | 78.00% |
| **500-999** | 73.98% | 74.26% | 74.57% | 74.27% |
| **1k-5k** | 79.02% | 79.22% | 79.76% | 79.33% |
| **5k-10k** | 72.76% | 71.36% | 73.77% | 72.63% |
| **>10k** | 76.13% | 76.12% | 79.90% | 77.38% |
| **Deletion** | 84.13% | 84.44% | 83.51% | 84.02% |
| **Insertion** | 73.55% | 73.93% | 74.28% | 73.92% |
| **Duplication** | 56.11% | 54.88% | 58.90% | 56.63% |
| **Inversion** | 50.13% | 48.47% | 52.87% | 50.49% |
| **Total-GT** | 70.67% | 71.23% | 73.05% | **71.65%** |
| **Average-MCC** | | | | |
| **Total-MCC** | 50.21% | 53.73% | 54.63% | 52.86% |
| **Average-Recall** | | | | |
| **Total-Recall** | 70.29% | 70.41% | 69.06% | 69.92% |
| **Average-Precision** | | | | |
| **Total-Precision** | 85.94% | 86.05% | 88.18% | 86.73% |

**Note:** All the numbers in bold are mentioned in the main article.

**Table S13. Detailed description of SV callers.**

| **Tool** | **Programming Language** | **SV type** | **Multi-thread** | **Detailed setting** | **Version** | **Availability** |
| --- | --- | --- | --- | --- | --- | --- |
| cuteSV | Python3 | DEL, INS, INV, DUP, BND | Yes | cuteSV [BAM] [REF] [output] [work_folder] -s [num] -l 30 –genotype -mi 0 | 1.0.10 | <https://github.com/tjiangHIT/cuteSV> |
| PBSV | C++ | DEL, INS, INV, DUP, BND, CNV | Partial | pbsv discover [BAM] [svsig.gz] -s chm1 && pbsv call [REF] [svsig.gz] [output] | 2.3.0 | <https://github.com/PacificBiosciences/pbsv> |
| Sniffles | C++ | DEL, INS, INV, DUP, BND, INVDUP | Yes | sniffles -m [BAM] -v [output] -s [num] -l 30 --genotype | 1.0.12 | <https://github.com/fritzsedlazeck/Sniffles> |
| SVIM | Python3 | DEL, INS, INV, DUP, BND | No | svim alignment [work_folder] [BAM] [REF] –min_sv_size 30 --minimum_score 0 –minimum_depth 1 | 1.4.0 | <https://github.com/eldariont/svim> |
| NanoSV | Python3 | DEL, INS, DUP, BND | Yes | NanoSV [bam] -s Samtools -o [output] | 1.2.4 | <https://github.com/mroosmalen/nanosv> |
| NanoVar | Python3 | DEL, INS, INV, DUP, BND | Yes | nanovar [BAM] [REF] [work_folder] -x pacbio-clr | 1.3.8 | <https://github.com/cytham/nanovar> |
| SURVIVOR | C++ | DEL, INS, INV, DUP, BND | No | SURVIVOR merge [sample_file] 1000 2 1 0 0 30 [output] | 1.0.7 | https://github.com/fritzsedlazeck/SURVIVOR |
